# Autoimmune Thyroid Disease with Fluctuating Thyroid Function

**DOI:** 10.1371/journal.pmed.0020089

**Published:** 2005-05-31

**Authors:** Ali S Alzahrani, Saleh Aldasouqi, Suzan Abdel Salam, Ali Sultan

**Affiliations:** Department of Medicine, King Faisal Specialist Hospital and Research Centre, RiyadhSaudi Arabia

## Abstract

The authors describe the case of a woman with autoimmune thyroid disease, who presented with hypothyroidism but went on to develop hyperthyroidism.

## PRESENTATION OF CASE

A 38-y-old East Asian woman presented in 1985 with weight gain and cold intolerance. She was found to have a small goiter and an elevated thyroid-stimulating hormone (TSH) level of 58 mU/l (normal range, 0.5–5). She was diagnosed with primary hypothyroidism and was treated with levothyroxine (L-T4) 0.1 mg/d. Her symptoms promptly improved. Between 1985 and 1993, she felt generally well. Her adherence to treatment was variable, and when she was reviewed her TSH levels were raised on several occasions (in the range of 15–38 mU/l). In her past medical history, she had an ectopic pregnancy in 1990 and bilateral oophorectomy in 2000 for benign cystadenomas. There was no family history of thyroid or autoimmune diseases.

While the patient was on vacation in the summer of 1993, she stopped taking L-T4 altogether. Upon her return, thyroid function tests (TFTs) showed a TSH level of 0.46 mU/l, free thyroxine (FT4) at 15 pmol/l (normal range, 11–24), and thyroid hormone T3 at 1.9 nmol/l (normal range, 1.0–2.5). Antimicrosomal antibody titer was 1:400. Between June 1994 and November 1998, she continued to feel well on no treatment. TSH was measured annually and remained normal (range, 0.8–1.6 mU/l).

**Figure 1:**
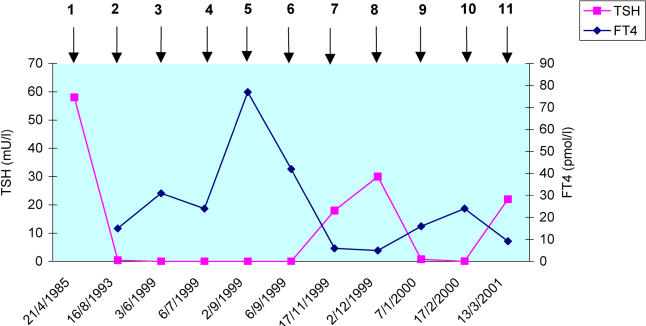
Alternating Thyroid Function over Time Numbered time points: (1) initial diagnosis of hypothyroidism; (2) off L-T4 for 5 wk while on vacation; (3) presentation with Graves' disease; (4) I^131^ treatment; (5) severe hyperthyroidism 5 wk after I^131^ treatment; (6) a few days after treatment with propylthiouracil, propranalol, and Lugol's solution; (7) hypothyroidism about 4 mo after I^131^ treatment off all medications; (8) hypothyroidism confirmed with another set of TFTs and L-T4 started; (9) relapse of hyperthyroidism and L-T4 discontinued; (10) hyperthyroidism confirmed and methimazole started; (11) hypothyroidism developed and L-T4 resumed.

On 3 June 1999, the patient presented with palpitations, heat intolerance, excessive sweating, and anxiety. She also reported eye staring but no diplopia, proptosis (bulging eyes), or eye pain. She had no neck pain, dysphagia, or change in voice. She was clinically thyrotoxic with tachycardia, tremor, lid lag, and retraction. She had a symmetrical goiter, 40 g in size, that was firm, non-tender, and mobile with no distinct nodules or bruits. Hertel exophthalmometric measurements were 18 mm in both eyes (normal is up to 18 mm). The rest of the examination was unremarkable. TSH was less than 0.06 mU/l and FT4 was 31 pmol/l (Figure 1). Liver enzymes were normal. Radioiodine uptake was 41% (normal range, 25%–35%) at 24 h with homogeneous distribution of the tracer.

On 8 June 1999, the patient was started on methimazole 20 mg/d. By 2 July 1999, she was feeling well, and TFTs showed FT4 at 24 pmol/l, T3 at 2.2 nmol/l, and TSH at less than 0.06 mU/l. On 25 July 1999, she was treated with 14 mCi of I^131^. On 2 September 1999, the patient presented with severe symptoms of palpitations, heat intolerance, tremor, headache, nausea, and vomiting. Clinically, she was severely thyrotoxic, with a regular pulse of 110/min, and an oral temperature of 37.4 °C. She was irritable but fully conscious and oriented. She had lid lag and retraction and mild proptosis of the left eye (20 mm). The goiter was the same size (40 g) and consistency as during her presentation on 3 June1999, but it was now moderately tender. TFTs showed FT4 at more than 77 pmol/l, T3 at 6.4 nmol/l, and TSH at less than 0.06 mU/l. Methimazole was increased to 15 mg three times a day. Because of the severity of her hyperthyroidism, Lugol's solution was added at five drops, three times a day, for 14 d. The patient declined corticosteroids.

After a few days, she felt better and repeat FT4 was 42 pmol/l and TSH was less than 0.06 mU/l. Liver enzymes, however, were now raised: AST at 95 U/l, ALP at 372 U/l, and total bilirubin at 10 μmol/l. The methimazole was discontinued and replaced 2 wk later with propylthiouracil at a dose of 100 mg twice daily. The patient continued to improve over the following several weeks. Liver enzymes slowly normalized after about 2.5 mo. On 3 November 1999, the propylthiouracil was discontinued. TFTs done 2 and 4 wk later showed FT4 at 6 and 5 pmol/l and TSH at 18 and 30 mU/l, respectively. It was thought that this might be a transient hypothyroidism after I^131^ therapy or a permanent post-ablation hypothyroidism. The latter was considered more likely, and therefore the patient was started on L-T4. Five weeks later, she was doing well, and was clinically euthyroid with FT4 of 16 pmol/l and TSH of 0.8 mU/l.

In February 2000, the patient presented with symptoms of hyperthyroidism and her TSH was low, at 0.05 mU/l. L-T4 was discontinued, but she remained hyperthyroid 2 mo later. She was then started on methimazole 10 mg once daily. In May 2000, a repeat 24-h radioiodine uptake was elevated at 65%. The patient was continued on methimazole until December 2000, when the drug was discontinued because the patient was found to be euthyroid.

She remained euthyroid off all treatment for 2 mo, but in March 2001, she started to have symptoms of hypothyroidism and her TSH rose to 22 mU/l, with a FT4 of 9.2 pmol/l. She was started on L-T4 50 μg once daily, on which she remained euthyroid with repeatedly normal TFTs. Between March 2001 and December 2004, she remained euthyroid on L-T4 50 μg once daily (TSH ranged between 1.2 and 2.8 mU/l). She was last seen on 11 December 2004, when she was clinically and biochemically euthyroid with FT4 of 18.4 pmol/l and TSH of 2.8 mU/l.

## DISCUSSION

The two main disorders that comprise autoimmune thyroid disease are Hashimoto thyroiditis and Graves disease. The former is the most common cause of hypothyroidism, whereas the latter is a major cause of hyperthyroidism. Occasionally, a patient may present with features of one of these disorders at one time and features of the other at another time. The usual sequence is hyperthyroidism followed by hypothyroidism [1]. However, cases of hypothyroidism followed by hyperthyroidism, such as in our patient, have also been described [2]. Our patient developed Graves disease after many years of hypothyroidism followed by a similar period of euthyroidism. The initial phase of hypothyroidism was secondary to Hashimoto thyroiditis since the patient had goiter and high antimicrosomal antibodies. The hypothyroidism did not represent a hypothyroid phase of an episode of thyroiditis since the patient repeatedly had elevated TSH levels over several years when she was not adhering to L-T4 therapy. Interestingly, following treatment with I^131^, she developed multiple alternating phases of hypothyroidism and hyperthyroidism (Figure 1). It is possible that the thyroiditis caused by I^131^ induced some immune reaction, with the formation of stimulating and inhibiting antibodies leading later to alternating phases of hypo- and hyperthyroidism.

Takasu et al. described eight cases of autoimmune thyroid disease with an alternating pattern of thyroid function [2]. In five cases, hypothyroidism was followed by transient hyperthyroidism. In two other cases, hypothyroidism was followed by persistent hyperthyroidism, and in one case hypothyroidism was associated with thyroid-stimulating antibodies, a characteristic finding in Graves disease. Tamai et al. reported the development of spontaneous hypothyroidism in 26 patients with Graves disease treated with antithyroidal drugs [1]. In another reported case, a patient underwent three cycles of transition from hypo- to hyperthyroidism and back to hypothyroidism, with corresponding changes in stimulating and blocking TSH receptor antibodies [3]. In our patient, we did not have serial measurements of stimulating and blocking thyroid antibodies. However, it is likely that the alternating thyroid function was associated with an alternating pattern of antibodies.

Recovery from hypothyroidism in chronic autoimmune thyroiditis is a rare but recognized phenomenon [4]. This is more likely to happen after iodide restriction in countries with high dietary iodide intake [5]. Predictors of spontaneous recovery from hypothyroidism in Hashimoto thyroiditis include the presence of goiter and high radioiodine uptake [5–7]. Spontaneous recovery also has been related to the disappearance of TSH-receptor-blocking antibodies [8].

Our patient had severe hyperthyroidism 5 wk after I^131^ treatment, while she was taking a relatively large dose of methimazole. This was most likely secondary to I^131^-induced thyroiditis as there was tenderness of the thyroid gland and FT4 increased proportionally much more than T3. The use of thionamide drugs to establish euthyroidism prior to I^131^ treatment is controversial, especially in a young patient without cardiovascular disease [9,10]. While rare, severe hyperthyroidism and even thyroid storm have been described following I^131^ treatment of Graves disease [11].

The patient also developed elevated liver enzymes. This could be secondary to the severe hyperthyroidism per se or due to the high dose of methimazole. Elevated liver enzymes are occasionally seen as a manifestation of hyperthyroidism and are also a well-recognized complication of antithyroid drugs [12–14].

**Learning Points**

• Rarely, autoimmune hyperthyroidism can occur after many years of hypothyroidism in patients with Hashimoto thyroiditis.

• When a patient on L-T4 for Hashimoto hypothyroidism presents with hyperthyroidism, over-replacement with L-T4 is the likely cause, but the possibility of endogenous hyperthyroidism should also be considered.

• Although radioactive iodine therapy for Graves disease is generally safe, especially in an otherwise healthy patient, severe exacerbations of hyperthyroidism can occur, and close monitoring in the first few weeks after therapy is prudent.

## Abbreviations

FT4: free thyroxine
L-T4: levothyroxine
TFT: thyroid function test
TSH: thyroid-stimulating hormone
